# Cytochrome C as a potential clinical marker for diagnosis and treatment of glioma

**DOI:** 10.3389/fonc.2022.960787

**Published:** 2022-09-13

**Authors:** Rashmi Rana, Rohit Singh Huirem, Ravi Kant, Kirti Chauhan, Swati Sharma, M. H. Yashavarddhan, Satnam Singh Chhabra, Rajesh Acharya, Samir Kumar Kalra, Anshul Gupta, Sunila Jain, Nirmal Kumar Ganguly

**Affiliations:** ^1^ Department of Research, Sir Ganga Ram Hospital, New Delhi, India; ^2^ Department of Neurosurgery, Sir Ganga Ram Hospital, New Delhi, India; ^3^ Department of Histopathology, Sir Ganga Ram Hospital, New Delhi, India

**Keywords:** cancer, glioma, cytochrome C, apoptosis, apoptotic protein array

## Abstract

Gliomas are the most prevalent kind of malignant and severe brain cancer. Apoptosis regulating mechanisms are disturbed in malignant gliomas, as they are in added forms of malignancy. Understanding apoptosis and other associated processes are thought to be critical for understanding the origins of malignant tumors and designing anti-cancerous drugs for the treatment. The purpose of this study was to evaluate the variation in the expression level of several apoptotic proteins that are responsible for apoptosis in low to high-grade glioma. This suggests a significant change in the expression of five apoptotic proteins: Clusterin, HSP27, Catalase, Cytochrome C, and SMAC. Cytochrome C, one of the five substantially altered proteins, is a crucial component of the apoptotic cascade. The complex enzyme Cytochrome C is involved in metabolic pathways such as respiration and cell death. The results demonstrated that Cytochrome C expression levels are lower in glioma tissues than in normal tissues. What’s more intriguing is that the expression level decreases with an increase in glioma grades. As a result, the discovery shows that Cytochrome C may be a target for glioma prognostic biomarkers.

## Graphical Abstract

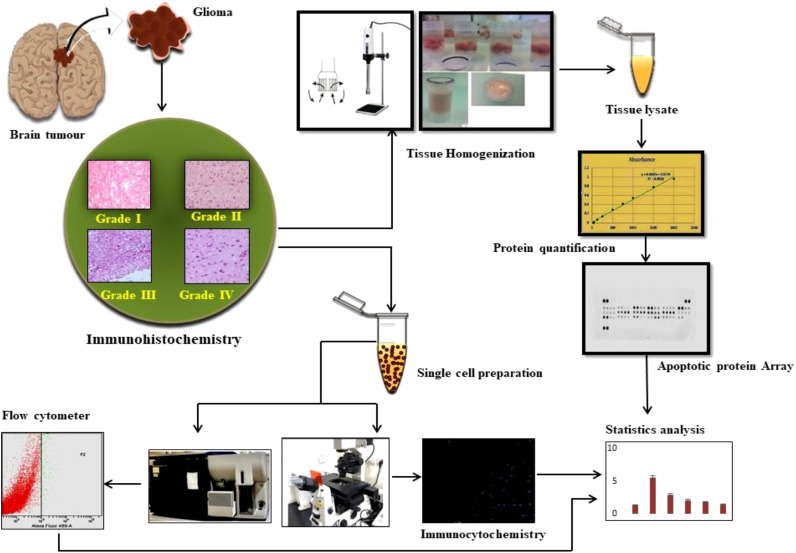

## Introduction

Glioma is the most frequent malignant primary brain tumor. Molecular biomarkers of various tumor types were revised in the World Health Organization (WHO) Central Nervous System (CNS) in 2021, based on the previous histological classification of gliomas from grade I to IV in the 2016 WHO classification and offer greater advantages and significant directions to clinicians (1). Neurons, which communicate with the nervous system, and glia (or glial cells or neuroglia), which provide support and protection to neurons, maintain homeostasis, and form myelin sheaths around them, are both implicated in gliomas and comprise the CNS (2). Glioblastoma (GBM) is one of the most devastating and recurrence-prone malignant solid tumors, accounting for 57% of all gliomas and 48% of primary CNS malignant tumors; it is regarded as one of the most catastrophic cancers due to its locally aggressive behavior and inability to be successfully treated by available treatments (3, 4). Gliomas remain obstinate to radiotherapy, surgery, immunotherapy, and chemotherapy. Cell proliferation, angiogenesis, migration, invasion, apoptosis, and autophagy are all regulated by different signaling pathways (5–7). Apart from IDH and MGMT, methylation has been considered an important prognostic biomarker. Other CNS biomarkers, such as TERT promoter mutation, EGFR amplification or mutation, and so on, are only connected to grade and help predict prognosis. Only the EGFRvIII mutation is categorically clinically instructive (8). Substitution or combination of radiation with chemotherapy or target therapy or personalized treatment for various patients has been recommended in recent decades (9–11).

One of the major contributors to the resistance of GBM to chemotherapy and radiotherapy is the deregulation of cell death pathways such as autophagy and apoptosis (12), leading to an overexpression of anti-apoptotic proteins as well as decreased level of several pro-apoptotic proteins. Apoptosis is a type of controlled cell death that is energy-dependent. In recent years, apoptosis research has mostly focused on altering the respiratory chain of mitochondria rather than altering the nuclear structure. Cytochrome C (Cyto C) is an essential molecule in mitochondria-induced apoptosis and an important component of energy metabolism as a fundamental component of the respiratory chain (13, 14). Mitochondrial Cyto C has been demonstrated to serve a dual role in cellular energy metabolism and apoptosis. The involvement of Cyto C in apoptosis was initially hypothesized by Liu et al. (15). Once released into the cytoplasm, Cyto C engages with its adaptor molecule Apaf-1 to activate pro-caspase-9 in the context of ATP or dATP. Caspases-9 and 3 are activated by activated caspase-9, resulting in the molecular features of apoptosis known as the intrinsic mitochondria route (16). An important initial step in the apoptotic process is the release of Cyto C from the mitochondria to the cytoplasm. As a component of the mitochondrial electron transport chain, Cyto C plays an important role in electron transfer between complex III (ubiquinol: Cyto C oxidoreductase) and complex IV (cytochrome oxidase) (17). Cyto C is a mitochondrial biomarker that is discharged into the extracellular space as well as the circulation within 1 hour following apoptosis induced by permeabilization of damaged mitochondria (18). As a result, Cyto C is regarded as a key mediator and biomarker in mitochondria-mediated apoptosis. Literature has shown that serum Cyto C forecasts prognosis throughout cancer treatment, including leukemia and lung cancer, indicating that Cyto C may play a role in tumor formation and development (19–21). Nonetheless, the clinical significance and role of Cyto C in glioblastoma are unknown. This study elucidates the alterations in Cyto C, apoptotic pathway proteins in glioma.

## Materials and methods

### Reagents and samples

Pierce™ BCA Protein Assay Kit (Cat. No. 23225), was purchased from Thermo Fisher Scientific, Rockford, IL, USA. Human Apoptosis Array Kit of Proteome Profiler™ Array (Cat. No. ARY009) were procured from Bio-Techne, Minneapolis/St. Paul International Airport. Paraformaldehyde (Cat. No. 158127), Potassium Chloride (Cat. No. P9541), ammonium chloride (Cat. No. A9434), sodium chloride (Cat. No. S3014), potassium phosphate monobasic (Cat. No. P9791), tween 20 (Cat. No. P9416), bovine serum albumin (BSA) (Cat. No. 5482), triton X-100 (Cat. No. T8787), CHAPS hydrate (Cat. No. C3023- 5G), Protease Inhibitor Cocktail (Cat. No. P8340), Sodium Phosphate Dibasic, TRIZMA base (Cat. No. T1503), hematoxylin (Cat. No. 1051752500), eosin (Cat. No. E4382-100G) was purchased from Sigma–Aldrich, St. Louis, MO, United States. TrypLE ™ Express Enzyme (1X), no phenol red (Cat. No. 12604021), Cytochrome C Monoclonal Antibody (Cat. No. MA5-11674), Alexa Flour ™ 488 Goat anti-mouse IgG (H+L) (Cat. No. A-11001) were purchased from Thermo Fisher Scientific, United States. Urea (Cat. No. V3171), DAPI (Cat. No. F6057), DNeasy^R^ Blood & Tissue kit (Cat. No. 69504) were purchased from Qiagen, agarose (Cat. No. A9539-500G), Ethidium di-bromide (Cat. No. E8751-5G) from Sigma-Aldrich.

1 L 1X PBS (8 g of sodium chloride, 0.2 g of potassium chloride, 1.44 g of sodium phosphate dibasic, 0.25 g of potassium phosphate monobasic to 1 L at pH 7.4), 10% poly-L-lysine/PBS for slide coating, 0.1% PBST (0.1% tween 20 in PBS), 1% BSA/0.1% PBST, 70% ethanol in PBS, permeabilization buffer (0.25% of triton X-100 in wash buffer), Lysis Buffer (20Mm, 4% CHAPS, 8M urea), RNA lysis buffer (50 mM ammonium chloride (A9434), 1X PBS), 1L 1X TAE (40Mm Tris (7.6 pH), 20Mm acetic acid, 1mM EDTA).

### Patients recruitment

#### Inclusion criteria

This study covered patients who had been diagnosed with glioma tumors. For Healthy individuals, the sample was taken from the person who died in an accident with consent from his/her family. Details of the patients involved in the study are mentioned in **Supplementary Table 1**

#### Exclusion criteria

Patients under the age of 18 and those who refused to give consent were excluded from the study.

All tissue associated with the disease was obtained from patients during surgery, performed for the resection of the glioma, and consecutively operated on in the department of neurosurgery at Sir Ganga Ram Hospital-Delhi, India. These were the recently diagnosed patients whose clinical data and investigations were obtained from the clinicopathological referral sheets. Informed consent was obtained from each patient and protocols were approved by the Sir Ganga Ram Hospital, Human Ethical Committee (Ref no. EC/10/17/1270) Delhi, India. All experiments were carried out by relevant guidelines and regulations.

### Histopathological examination

All the experiment was standardized and optimize thrice for better reproducibility. The tissue specimens investigated in this research work consist of human biopsies extracted during brain tumor resection procedures hospitals. After the resection, the samples were dehydrated and embedded in paraffin blocks. The blocks were then mounted in microtomes and sliced into 4 µm thick slices. Finally, the slices were rehydrated and stained with H&E. After routine examination of the samples, every sample was diagnosed by pathologists as GBM, according to the World Health Organization (WHO 2016) classification of tumors of the nervous system. Photomicrographs were captured at a magnification of 20X. At the end of the examination, Grades were identified according to WHO guidelines.

### Genomic DNA extraction

Extraction of genomic DNA from glioma and healthy tissue were isolated using DNeasy^R^ Blood & Tissue kit. All reagents and samples were prepared according to the Kit. The assay was performed according to the protocol mentioned in the kit. The extracted DNA was quantified using a spectrophotometer, NanoDrop 2000 (Thermo Scientific, USA). The DNA samples were electrophoresed on a 0.8% agarose gel. The gel was examined and photographed by iBright 1500(Invitrogen, Thermo fisher scientific, USA).

### Protein isolation and quantification

Extraction of protein from glioma and healthy tissue is essential to make the entire protein complement accessible for further studies. Protein was extracted as described previously (22). With few modifications. For protein extraction, 150 mg of tissue of each grade and a healthy sample were taken and washed with 1X Phosphate buffer saline (PBS). After washing the samples, 2ml of lysis buffer was added. 1µl of Protease Inhibitor Cocktail was also added. Further, homogenization of the sample was done (3 cycles; 30 seconds on and 10 sec off for each cycle). After homogenization, the homogenate was centrifuged at 15000 rpm for 30 minutes, 4°C. Filter the Supernatant with a 0.22-micron filter membrane and discard the pellets. Concentrations of the proteins in samples were determined by using the Bicinchoninic acid assay (BSA) based on the manufacturer’s protocol (Pierce™ BCA Protein Assay Kit Cat. No. 23225).

### Human apoptosis array

To determine expression level changes in different proteins responsible for altered levels of apoptosis in low-grade glioma to higher-grade glioma Human Apoptosis Array Kit of Proteome Profiler™ Array (Cat. No. ARY009) was used. All reagents and samples were prepared according to the Array Kit (Cat. No. ARY009). The assay was performed according to the protocol mentioned in the kit.

### Single-cell suspension preparation from tissue

Single-cell suspensions were made as previously described (23), with minor modifications based on our tissue. Glioma tissues were washed using phosphate-buffered saline and cut into small pieces. It was immersed into TrypLE ™ Express Enzyme (1X), with no phenol red (Cat. No. 12604021) for 10 min, 37 °C. Then it was digested by mechanical means. Cells were filtered using a cell strainer (100micrometer) and cell pellets were collected by centrifuging 2000rpm for 10min. Then pellet was washed twice using phosphate-buffered saline. Next, cells were counted using a hemocytometer.

### Immunocytochemistry study

The study’s goal was to determine the degree of Cyto C expression in different grades of Glioma tissue samples. Tissues were washed in PBS, and single-cell suspensions from tissues were prepared mechanically. As previously stated, the samples were (24). Cells were fixed in 4 percent formaldehyde/PBS for 30 minutes on ice before being spotted onto Poly-L-Lysine coated glass at a concentration of 1 105 cells/ml. Unlysed RBCs were extracted with 50 mM ammonium chloride. Following that, cells were treated for 1 hour in 1 percent BSA in 0.1 percent PBS-Tween to permeabilize the cells and prevent non-specific protein-protein interactions. Following blocking, cells were treated overnight at 4α with Cyto C (2ug/ml). Cells were washed and incubated in 1 percent BSA for 1 hour with Alexa Flour ™488 Goat anti-mouse IgG (H+L) (1ug/ml). After washing, the slides were incubated with DAPI at room temperature for 30 minutes. After that, the slides were cleaned, dried, and mounted. Images were captured at 10x magnification using a fluorescence microscope (Nikon eclipse Ti).

### Flow cytometric measurement of Cyto C

Flow cytometric measurements were utilized to confirm the Cyto C data acquired from the immunocytochemistry investigation. PBS was used to wash the tissue, and an enzymatic and mechanical method was used to prepare a single cell suspension. Glioma cells were washed twice with PBS (centrifugation at 1200 rpm for 10 minutes) before being fixed in 100 l of 3% paraformaldehyde for 30 minutes on ice. The fixed cells were rinsed twice with 1 ml of PBS, then with 1 ml of 50 mM ammonium chloride. After two PBS washes, the cells were permeabilized in 200 l of 0.1 percent Triton X-100 in PBS for 20 minutes at 4°C. The blocking step was performed in 10% BSA in PBS for 100 min at room temperature, followed by overnight incubation with Cyto C Monoclonal Antibody (5ug/ml) as per the experimental design. The cells were then washed with PBS and incubated with Alexa Flour™ 488 Goat anti-mouse IgG (H+L) (5ug/ml) at 4°C. Data were acquired in a conventional flow cytometer (FACS Aria BD Biosciences, San Jose, CA, USA).

### Bioactivity assay

Using PubChem (https://pubchem.ncbi.nlm.nih.gov/) we have searched for the interaction of the drug with Cyto C with a publicly available database.

### Statistical analysis

A laboratory register was used to record results and results were also reproduced into a spreadsheet of SPSS 17.0. The intensities were quantified in different grades of glioma in comparison to healthy control using Image J software (NIH). Comparisons were made among the different grades against the control for immunocytochemistry, protein array, and FACS. The data obtained are represented as mean ± SD. The difference between the experimental groups was evaluated by one-way analysis of variance, with Newman-Keuls multiple comparison tests (V, 5.01; Graph Pad Prism, San Diego, CA, United States. Comparisons among the healthy individuals and different grades of Gliomas for immunocytochemistry and FACS were done.

## Results

### Histopathological analysis of different grades of glioma

**Figure 1:**
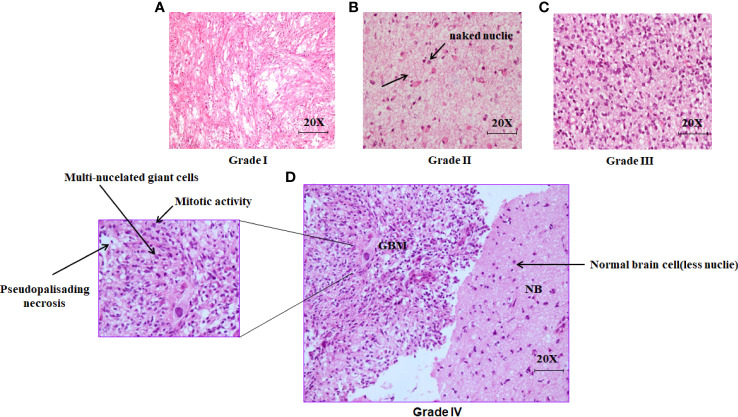
Histopathological analysis of excised brain tumor tissue stained with hematoxylin and eosin (H&E). **(A)** The light microscopic picture depicts the glioma grade I under 20X magnification. **(B)** The Light microscopic picture depicts the glioma grade II under 20X magnification with the formation of naked nuclei. **(C)** The light microscopic picture depicts the glioma grade III under 20X magnification. **(D)** The light microscopic picture depicts the glioma grade IV (GBM) along with one portion of normal brain tissue in the right under 20X magnification.

The grade of Glioma was characterized by the cell proliferation rate, cell morphology along with their necrosis property. Pseudopalisading necrosis is highly observed in GBM (Grade IV) along with the giant cell with large, highly pleomorphic, multinucleated giant cells. However, In the case of other grades, it was noticed that there was a presence of naked nuclei. The presence of necrosis and/or multivariate proliferation (MVP) leads to a diagnosis of glioblastoma. The numbers of nuclei (blue) are higher in higher grades of glioma, indicating a higher rate of cell proliferation (**Figure 1** **).**

#### DNA fragmentation assay

**Figure 2:**
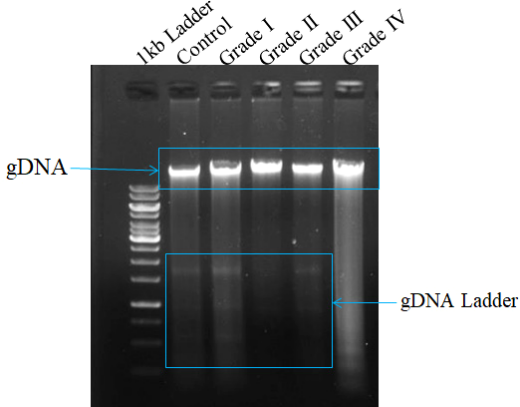
Measurement of apoptosis in different grades of glioma with comparison to control tissue on surgically excised tissue sample using DNA ladder assay. The formation of DNA fragments in the form of a ladder describes the occurrence of apoptosis.

Apoptosis can be visualized as a ladder pattern of 180-200bp due to DNA cleavage by the activation of nuclear endonucleases by standard agarose gel electrophoresis. Thus, we show the higher form of the DNA ladder in gel in the case of the healthy sample when compared to different grades. However, we witness a lower amount of DNA ladder in the case of Grade IV when compared among the different grades of glioma (**Figure 2**).

### Expression of different apoptotic proteins among different grades of glioma

**Figure 3:**
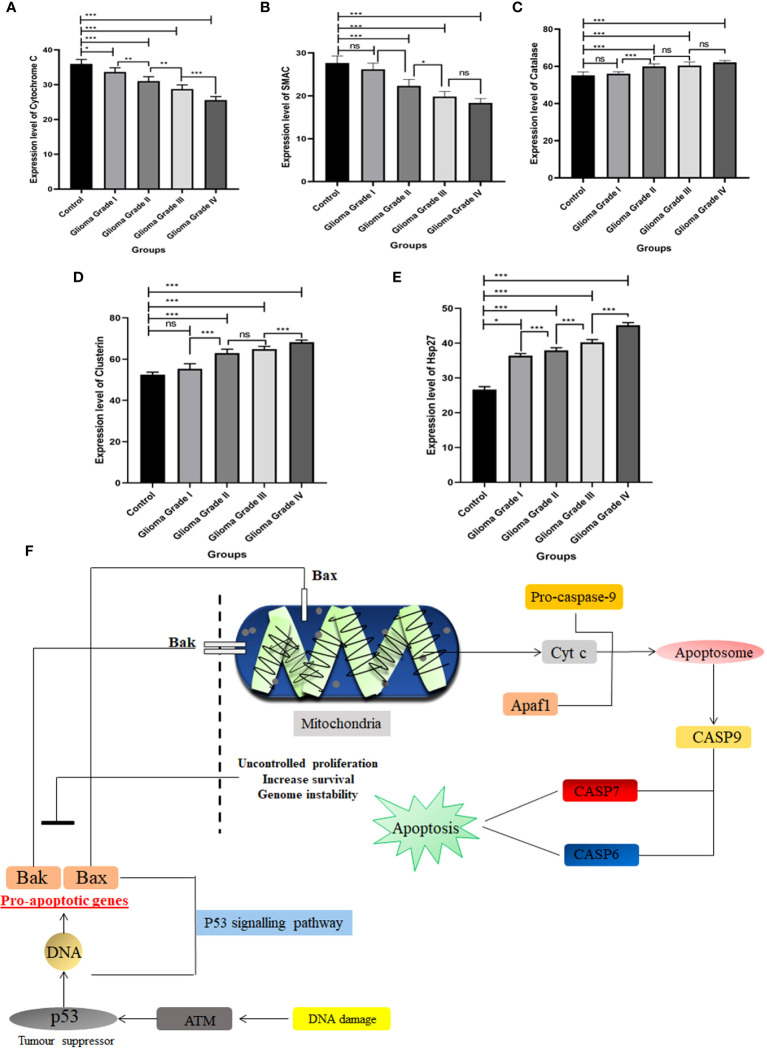
Expression pattern of different apoptosis proteins identified in Human Apoptosis Array. Total protein samples isolated from different grades of gliomas along with normal brain tissue were analyzed using a human apoptosis array kit. **(A)** Expression level of Cytochrome C gets down-regulated among different grades of Glioma with compared to control. **(B)** SMAC expression level gets down-regulated in different grades of Glioma in contrast to control.**(C)** Catalase expression increases in different grades of Glioma. **(D)** Expression of Clusterin protein among the different grades with contrast to control. **(E)** Hsp27 expression pattern in different grades of Glioma with compared to control. **(F)** Schematic representation of Cyto C pathway in apoptosis w.r.t Glioma. Cyto C activates apoptosis through the caspase pathway. **n**=6 per group. A value of *p < 0.05, **p < 0.01, ***p < 0.001, ^ns^ not-significant (p > 0.05).

The apoptotic potential of human glioma cells with different grades was analyzed using Image J software. The Human Apoptosis Array Kit of Proteome Profiler TM Array was used to identify expression level changes in different proteins in the apoptosis pathway, and membranes were then put in an iBright™, and spots were observed at different time intervals. Spots collected at 3 minutes were used to compare protein expression in all samples. Apoptotic arrays were used for normal individuals and patients with grade I, grade II, grade III, and grade IV glioma to determine the apoptotic potential of human glioma cells. The expression of three separate apoptotic genes rises as grades rise in comparison to normal individuals. On using Image J software, proteins such as Clusterin, HSP27, and Catalase expression level goes up and two proteins’ expression gets down-regulated when compared to control. Cyto C and SMAC are the two proteins whose expression gets down-regulated with the severity of human glioma. However, among all these five proteins the most significant was found to be Cyto C in contrast to healthy individuals as well among the different grades. We have done pathway analysis using KEGG Pathway, where we have merged two different pathways one which is involved in Glioma progression and another one is of the apoptosis pathway. We have proposed a connection between Cyto C in Glioma progression (**Figure 3**).

### Immunocytochemistry analysis of Cyto C in different grades of glioma

**Figure 4:**
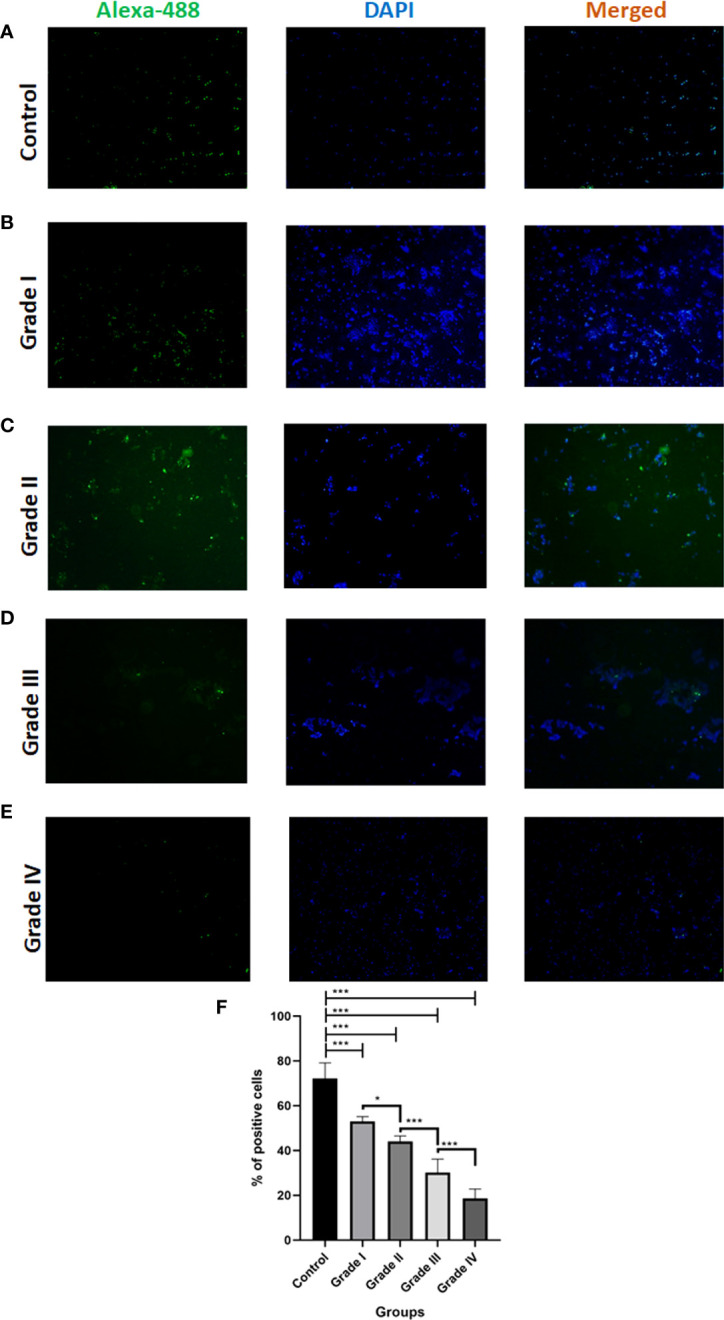
Immunocytochemistry analysis of Cytochrome C in different grades of glioma in comparison to control. **(A-E)** Representative fluorescent microscopic images depict the expression of apoptotic protein Cytochrome C along with nuclear dye DAPI expression in different grades of Glioma. **(F)** Graph represents the total % of Cytochrome C positive cells. n=6 per group. A value of *p < 0.05, ***p < 0.001.

To determine the Cytochrome C status in Glioma tissues and corresponding normal tissues were examined using immunocytochemistry. We notice that the proportion of total % Cyto C high positive cells in normal tissue (control) was found to be 72%, in the case of grade I, it was observed that the number of total positive cells was significantly reduced by 19% in comparison to control i.e., 53% whereas among the different grades it was observed that Grade I shows the higher the expression of positive cells. Cytochrome C protein expression levels were found to be lower in Glioma tissues than in normal tissues, and the number of positive cells declined as the grade of Glioma rose (Grade I 53% ± 2.10, Grade II 44% ± 2.45, Grade III 31% ± 6.10, and Grade IV 18% ± 4.16). Overall, these data suggest that low expression of Cytochrome C may be associated with glioma progression (**Figure 4** **).**

### Flow cytometry analysis for expression of Cyto C among different grades of glioma

To analyze the expression pattern of Cyto C among the different grades in healthy individuals, we measure the % of positive cells and mean fluorescent intensity (MFI) using Flow-Cytometry. As the severity of glioma increases, the expression of Cyto C decreases. In the case of control (healthy individual), it was observed that the positive cells % was 5.5 ± 0.1% and in comparison, to a different grade of glioma, it was noticed that the number of the positive cell was decreased (Grade I- 2.9 ± 0.3, Grade II-2.1 ± 0.4, Grade III-1.8 ± 0.1, Grade IV- 1.4 ± 0.2).

**Figure 5:**
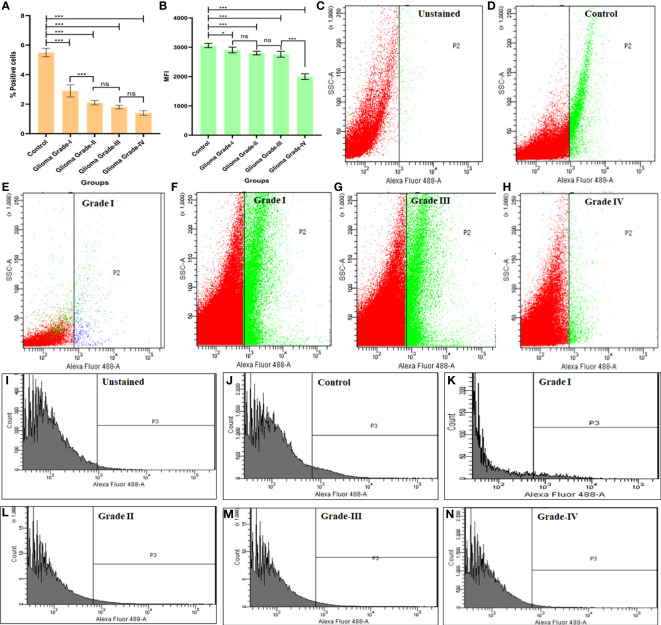
Flowcytometric measurement of cytochrome C protein in different grades of Glioma. **(A)** Graph represents % positive cells in different grades of glioma in comparison to normal brain cells. **(B)** Graph represents the mean fluorescent intensity (MFI) of Cyto C in different grades of glioma in comparison to normal brain cells. **(C-H)** Dot plot describes changes in expression of apoptotic protein Cyto C in different grades of glioma in comparison to normal brain cells. **(I-N)** Histogram represents changes in expression of Cyto C protein in different grades of glioma in comparison to normal brain cells. n=6 per group. A value of *p < 0.05, ***p < 0.001, ^ns^ not-significant(p > 0.05).

Here, we also measured the mean fluorescent intensity, and a similar pattern was observed (Control 3058 ± 73.4, Grade I 2906 ± 97.4, Grade II 2801 ± 62.0, Grade III 2760 ± 100.5, Grade IV 1997 ± 97.0) (**Figure 5** **).**

### Bioactivity assay of anti-cancer drugs for activation of Cyto C

**Figure 6:**
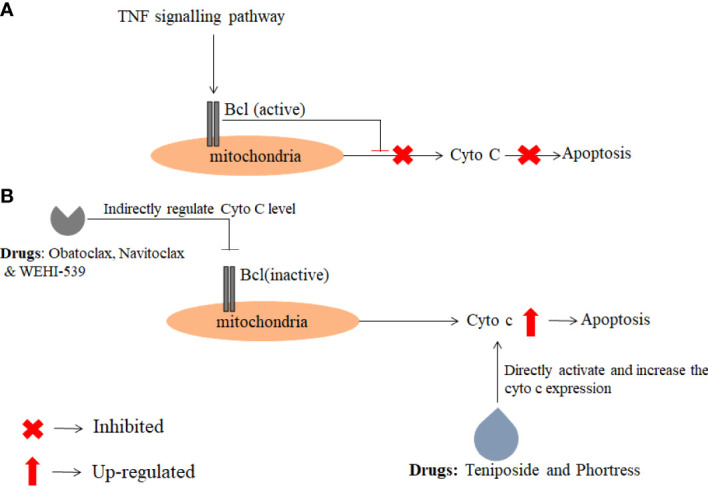
The figure depicts the action mechanism of anti-cancer drugs which regulate directly or indirectly Cyto C expression. The data were obtained from the PubChem database and a pathway was analyzed using KEGG pathway analysis. **(A)** Describes the inhibition of Cyto C expression in cancer conditions **(B)** Activation of Cyto C by anti-cancer drugs.

After analysis of drug interaction from the publicly available database, it was revealed that drugs such as Teniposide (PubChem CID: 452548), Phortress (PubChem CID: 399465), WEHI-539(PubChem CID: 71297207), Obatoclax (PubChem CID: 11404337) and Navitoclax (PubChem CID: 24978538) are directly or indirectly involved in regulating the expression of Cyto C **(** **Table 1** **).** Among these five drugs, two drugs i.e. Teniposide (PubChem CID: 452548), and Phortress (PubChem CID: 399465) directly enhance the expression of Cyto C which further leads to apoptosis. The targeted delivery of these two drugs could be potential therapeutic drugs for the treatment of glioma (**Figure 6** **).**

**Table 1:** List of Predicted drugs for inducing the expression of Cyto C w.r.t Glioma.

Compound name	FDA Approved	Compound class	Target
WEHI-539	No	Benzothiazole-hydrazone	BCL-X(L)
Teniposide	Yes	Podophyllotoxin derivatives	Cyto C
Phortress	No	Lysyl amide	Cyto C
Obatoclax	Yes	Dipyrrins	BCL
Navitoclax	Yes	Phenylpiperazines	BCL

## Discussion

Cancer cells vary from normal cells in several aspects, including autocrine growth signal generation, prolonged angiogenesis, the inability to respond to anti-growth signals, infinite replicative capability, apoptosis avoidance, tissue invasion, and metastasis. The discovery of useful biomarkers for cancer detection and diagnosis might be a positive step forward in cancer treatment. Apoptosis is required for tissue stability and has been related to several diseases, including cancer (25). The study of changes in the expression levels of various proteins may aid in the creation of new anticancer medications for tumor therapy. As indicated in our results section, we found five apoptotic proteins: Clusterin, catalase, Hsp27, SMAC, and Cyto C.

Clusterin has been characterized as a sensitive biomarker in intestinal tumors and found increased in higher grades with a frequency of 59.1% in comparison to low-grade tumor with a frequency of 32.6% (26, 27). Studies reported alterations in the expression of Clusterin at transcription and translational levels in pituitary adenomas with the help of microarray and LC-MS-MS (28). Several research has gathered evidence that changes in Clusterin expression may be directly associated with tumor metastasis. Clusterin is involved in a variety of processes, including adhesion, tissue remodeling, apoptosis, and cancer spread (29, 30)..

Resistance to apoptosis *via* triggering intercellular ROS signaling *via* membrane-associated catalase owing to decomposing hydrogen peroxide, peroxynitrite, and oxidizing NO appears to be one of the characteristics of tumor growth. These basic features of oncogenesis are the resistance of tumor cells to intercellular ROS signaling through membrane-associated catalase expression (31–34). Galina Deichman’s group discovered a link between Catalase-mediated protection of tumor cells from apoptotic ROS signaling and the H2O2-catabolizing phenotype (35) and confirmed that all transformed, and tumor cells produced significant amounts of extracellular superoxide anion, but the tumor cells were protected from ROS-mediated apoptosis induction *via* membrane-associated catalase expression.

Hsp27 generally present in a low amount in unstressed cells belongs to a family of small heat shock proteins present in all organisms (36). Experiments have demonstrated that Hsp72 or Hsp27 increase cell survival in response to apoptotic stimuli. Hsp72 has been reported to inhibit apoptosis through direct interaction with Apaf-1, thereby preventing the docking of pro-caspase-9 and its subsequent activation (37, 38). T98G and MOGGCCM cells are very sensitive to apoptosis induction by temozolomide and quercetin therapy when Hsp27 and Hsp72 expression is silenced, and programmed cell death is started by an internal signal (39). HSP abnormal expression has been found in numerous tumor forms, indicating that distinct HSPs have varying prognostic implications for different malignancies. HSP27 expression was shown to be downregulated in human low-grade glioma tissues (HGTs) compared to autologous para-cancerous brain tissues (PBTs), and it displayed a temporal and spatial change *in vitro* under heat shock treatment (43 degrees C/0-3 h). The rapid overexpression of HSP27 was most likely associated with the transient resistance to heat shock required for human glioma cell survival (40)

Studies also reported that SMAC interacts with the BIR2 and BIR3 domains of XIAP and release caspase-3 and caspase-9 respectively (41). SMAC overexpression results in apoptotic death of neoplastic cells due to the induction of caspase-3 triggered by Cyto-C (42, 43). These findings provoked the development of peptides derived from the amino-terminal of SMAC and function as therapeutic agents to induce death or to increase the apoptotic effect of chemotherapeutic agents. Cells with lower SMAC have higher apoptosis resistance which might be a major cause of cancer progression.

Evading apoptosis is one of cancer’s primary characteristics. Tumors initiation, development, and metastasis are caused by oncogenic events such as down-regulated tumor suppressor genes or up-regulated oncogenes that disturb apoptosis (44). The mitochondrial signaling system is involved in the release of Cyto C and apoptosis-inducing proteins from mitochondria *via* the Bcl-2/Bax axis, therefore triggering subsequent apoptotic executors (45–47). As a result, Cyto C might have a role in cancer start and progression. Although serum Cyto C is an accurate indication of cell death and decreased tumor growth during the first cycle of chemotherapy, the significance and role of Cyto C in Glioma before chemotherapy has yet to be determined to the best of our knowledge (48). One of the studies found that Cyto C protein expression levels in CCRCC tissues were lower than in normal tissues. Cyto C overexpression efficiently decreased CCRCC cell growth and caused cell death, but Cyto C knockdown reversed these effects (49). Combining clinically employed anticancer drugs (doxorubicin, paclitaxel, oxaliplatin, vinblastine, and vincristine) with Cyto C Decorated Hybrid Nanoparticles for Liver Cancer Therapy considerably improves apoptosis within cell lines, leading to cellular death. As a result, this combination strategy may offer the potential for future treatment protocols (50). In the present study, Cyto C expression was demonstrated to be decreased protein levels in Glioma tissues, which suggested that decreased Cyto C may be involved in Glioma tumorigenesis.

## Data availability statement

The raw data supporting the conclusions of this article will be made available by the authors, without undue reservation.

## Ethics statement

The studies involving human participants were reviewed and approved by Sir Ganga Ram Hospital, Human Ethical Committee (Ref no. EC/10/17/1270). Delhi, India. The patients/participants provided their written informed consent to participate in this study.

## Author contributions

RR contributed to the study design and concept. RR, RH, RK, and KC were involved in the study organization. RR, RH, KC, SS, RK, SSC, RA, SK, and AG contributed to the recruitment of patients and collection of data. RR, RH, MY, RK, and KC contributed to data analysis and data interpretation. RR, RH, MY, KC, RK, and SS wrote the manuscript. SJ performed histopathological experiments. RR and NG – elaboration of the final version of the manuscript, correction of the language, analysis of the data, and revised critically the work. All authors contributed to the article and approved the submitted version.

## Funding

This work was funded by the Indian Council of Medical Research Government of India under the Grant-in-aid scheme of the Department of Health Research. (R 1101/26/2021-GIA/HR)

## Acknowledgments

The authors are thankful to Sir Ganga Ram Hospital, Delhi, India, and Department of Health and Research (DHR), Government of India for providing the funding and necessary support. Authors are sincerely thankful to Mr. Kennedy Roy, English Teacher, Victoria Boys’ School, West Bengal and Ms. Priyadarshini Moirangthem Chanu, Lecturer of English, MECI Explorer Academy, Manipur for their contribution in editing and making this research paper errorless.

## Conflict of interest

The authors declare that the research was conducted in the absence of any commercial or financial relationships that could be construed as a potential conflict of interest.

## Publisher’s note

All claims expressed in this article are solely those of the authors and do not necessarily represent those of their affiliated organizations, or those of the publisher, the editors and the reviewers. Any product that may be evaluated in this article, or claim that may be made by its manufacturer, is not guaranteed or endorsed by the publisher.

## References

[1] Louis DN Perry A Reifenberger G von Deimling A Figarella-Branger D Cavenee WK. The 2016 world health organization classification of tumors of the central nervous system: a summary. Acta Neuropathol (2016) 6:803–20. doi: 10.1007/s00401-016-1545-1 27157931

[2] Jessen KR Mirsky R. Glial cells in the enteric nervous system contain glial fibrillary acidic protein. Nature (1980) 286:736–7. doi: 10.1038/286736a0 6997753

[3] Ostrom QT Gittleman H Liao P Vecchione-Koval T Wolinsky Y Kruchko C. CBTRUS statistical report: primary brain and other central nervous system tumors diagnosed in the united states in 2010–2014. Neuro-oncology (2017) 19:88. doi: 10.1093/neuonc/nox158 PMC5693142 29117289

[4] Weller M Le Rhun E. How did lomustine become standard of care in recurrent glioblastoma? Cancer Treat Rev (2020) 87:102029. doi: 10.1016/j.ctrv.2020.102029 32408220

[5] Machein MR Plate KH. VEGF in brain tumors. J Neuro-Oncol (2000) 50:109–120. doi: 10.1023/a:1006416003964 11245271

[6] Katz AM Amankulor NM Pitter K Helmy K Squatrito M Holland EC. Astrocyte-specific expression patterns associated with the PDGF-induced glioma microenvironment. PloS One (2012) 7:e32453. doi: 10.1371/journal.pone.0032453 22393407 PMC3290579

[7] Voldborg BR Damstrup L Spang-Thomsen M Poulsen HS. Epidermal growth factor receptor (EGFR) and EGFR mutations, function and possible role in clinical trials. Ann Oncol (1997) 8:1197–206. doi: 10.1023/a:1008209720526 9496384

[8] Louis DN Perry A Wesseling P Brat DJ Cree IA Figarella-Branger D. The 2021 WHO classification of tumors of the central nervous system: A summary. Neuro-oncology (2021) 23:1231–51. doi: 10.1093/neuonc/noab106 PMC8328013 34185076

[9] Baumert BG Hegi ME Van Den Bent MJ Von Deimling A Gorlia T Hoang-Xuan K. Temozolomide chemotherapy versus radiotherapy in high-risk low-grade glioma (EORTC 22033– 26033): a randomised, open-label, phase 3 intergroup study. Lancet Oncol (2016) 17:1521–32. doi: 10.1093/neuonc/noab106 PMC5124485 27686946

[10] Buckner JC Shaw EG Pugh SL Chakravarti A Gilbert MR Barger GR. Radiation plus procarbazine, CCNU, and vincristine in low-grade glioma. N Engl J Med (2016) 374:1344–55. doi: 10.1056/NEJMoa1500925 PMC5170873 27050206

[11] Lassaletta A Scheinemann K Zelcer SM Hukin J Wilson BA Jabado N. Phase II weekly vinblastine for chemotherapy-naïve children with progressive low-grade glioma: A Canadian pediatric brain tumor consortium study. J Clin Oncol (2016) 34:3537–43. doi: 10.1200/JCO.2016.68.1585 27573663

[12] Kornienko A Mathieu V Rastogi SK Lefranc F Kiss R. Therapeutic agents triggering nonapoptotic cancer cell death. J Med.Chem (2013) 56:4823–4839. doi: 10.1021/jm400136m 23517069

[13] Zou H Li Y Liu X Wang X. An APAF-1 cytochrome c multimeric complex is a functional apoptosome that activates procaspase-9. J Biol Chem (1999) 274:11549–11556s. doi: 10.1074/jbc.274.17.11549 10206961

[14] Renault TT Floros KV Chipuk JE. BAK/BAX activation and cytochrome c release assays using isolated mitochondria. Methods (2013) 61:146–55. doi: 10.1016/j.ymeth.2013.03.030 PMC3686896 23567751

[15] Liu X Kim CN Yang J Jemmerson R Wang X. Induction of apoptotic program in cell-free extracts: requirement for dATP and cytochrome c. Cell (1996) 86:147–57. doi: 10.1016/s0092-8674(00)80085-9 8689682

[16] Robertson JD Orrenius S Zhivotovsky B. Review: nuclear events in apoptosis. J Struct Biol (2000) 129:346–58. doi: 10.1006/jsbi.2000.4254 10806085

[17] Cai JY Yang J Jones DP. Mitochondrial control of apoptosis: the role of cytochrome c. Biochim Biophys Acta (1998) 1366:139–49. doi: 10.1016/s0005-2728(98)00109-1 9714780

[18] Renz A Berdel WE Kreuter M Belka C Schulze-Osthoff K Los M. Rapid extracellular release of cytochrome c is specific for apoptosis and marks cell death in vivo. Blood.The J Am Soc Hematol (2001) 98:1542–8. doi: 10.1182/blood.v98.5.1542 11520805

[19] Wen Q Zhang X Cai J Yang PH. A novel strategy for real-time and in situ detection of cytochrome c and caspase-9 in hela cells during apoptosis. Analyst (2014) 139:2499–506. doi: 10.1039/c3an02205f 24665465

[20] Barczyk K Kreuter M Pryjma J Booy EP Maddika S Ghavami S. Serum cytochrome c indicates in vivo apoptosis and can serve as a prognostic marker during cancer therapy. Int J Cancer (2005) 116:167–73. doi: 10.1002/ijc.21037 15800951

[21] Osaka A Hasegawa H Tsuruda K Inokuchi N Yanagihara K Yamada Y. Serum cytochrome c to indicate the extent of ongoing tumor cell death. Int J Lab Hematol (2009) 31:307–14. doi: 10.1111/j.1751-553X.2008.01033 18279425

[22] Ericsson C Nistér M. Protein extraction from solid tissue. Methods Mol Biol (2011) 12:675–307. doi: 10.1007/978-1-59745-423-0_17 20949398

[23] Leelatian N Doxie DB Greenplate AR Sinnaeve J Ihrie RA Irish JM. Preparing viable single cells from human tissue and tumors for cytomic analysis. Curr Protoc Mol Biol (2017) 118:21–25C. doi: 10.1002/cpmb.37 PMC5518778 28369679

[24] Yashavarddhan MH Shukla Sandeep K Pankaj C Srivastava Nitya N Jayadev J Mrutyunjay S. Targeting DNA repair through podophyllotoxin and rutin formulation in hematopoietic radioprotection: An in silico, in vitro, and in vivo study. Front Pharmacol (2017) 8:1663–9812. doi: 10.3389/fphar.2017.00750 PMC5671582 29163150

[25] Camp RL Dolled-Filhart M Rimm DL. X-Tile: A new bio-informatics tool for biomarker assessment and outcome-based cut-point optimization. Clin Cancer Res (2004) 10:7252–9. doi: 10.1158/1078-0432.CCR-04-0713 15534099

[26] Shapiro B Tocci P Haase G Nancy G Avri BZ. Clusterin, a gene enriched in intestinal stem cells, is required for L1-mediated colon cancer metastasis. Oncotarget (2015) 6:34389–401. doi: 10.18632/oncotarget.5360 PMC4741460 26399194

[27] Chen X Halberg RB Ehrhardt WM Jose T William FD. Clusterin as a biomarker in murine and human intestinal neoplasia. Proc Natl Acad Sci USA (2003) 100:9530–5. doi: 10.1073/pnas.1233633100 PMC170952 12886021

[28] Yu SY Hong LC Feng J You TW Zhang YZ. Integrative proteomics and transcriptomics identify novel invasive-related biomarkers of non-functioning pituitary adenomas. Tumour Biol (2016) 37:8923–30. doi: 10.1007/s13277-015-4767-2 26753958

[29] Zhang F Kumano M Beraldi E Ladan F Caigan D Susan M. Clusterin facilitates stress-induced lipidation of LC3 and autophagosome biogenesis to enhance cancer cell survival. Nat Commun (2014) 5:5775. doi: 10.1038/ncomms6775 25503391 PMC4275590

[30] Lau SH Sham JS Xie D Tzang CH Tang D Ma N. Clusterin plays an important role in hepatocellular carcinoma metastasis. Oncogene (2006) 25:1242–50. doi: 10.1038/sj.onc.1209141 16247463

[31] Engelmann I Bauer G. How can tumor cells escape intercellular induction of apoptosis? Anticancer Res (2000) 20:2297–306. 10953288

[32] Engelmann I Dormann S Saran M Bauer G. Transformed target cell-derived superoxide anions drive apoptosis induction by myeloperoxidase. Redox Rep (2000) 5:207–14. doi: 10.1179/135100000101535762 10994875

[33] Bechtel W Bauer G. Catalase protects tumor cells against apoptosis induction by intercellular ROS signaling. Anticancer Res (2009) 29:4541–57. 20032403

[34] Bechtel W Bauer G. Modulation of intercellular ROS signaling of human tumor cells. Anticancer Res (2009) 29:4559–70. 20032404

[35] Arnold RS Shi J Murad E Whalen AM Sun CQ Polavarapu R. Hydrogen peroxide mediates the cell growth and transformation caused by the mitogenic oxidase Nox1. Proc Natl Acad Sci USA (2001) 98:5550–5. doi: 10.1073/pnas.101505898 PMC33250 11331784

[36] Arrigo AP Mehlen P. Expression and function of the low molecular weight heat shock proteins. In: Morimoto R Tisseres A, editors. The biology of heat shock proteins and molecular chaperones. New York: Cold Spring Harbor Laboratory press (1994). p. 335–73. Georgopoulos.

[37] Beere HM Wolf BB Cain K Helen MB Mosser DD Artin M. Heat- shock protein 70 inhibits apoptosis by preventing recruitment of procaspase-9 to the apaf-1 apoptosome. Nat Cell Biol (2000) 2:469–75. doi: 10.1038/35019501 10934466

[38] Saleh A Srinivasula SM Balkir L Robbins PD Alnemri ES. Negative regulation of the apaf-1 apoptosome by Hsp70. Nat Cell Biol (2000) 2 476–483. doi: 10.1038/35019510 10934467

[39] Jakubowicz-Gil J Langner E Bądziul D Wertel I Rzeski W. Silencing of Hsp27 and Hsp72 in glioma cells as a tool for programmed cell death induction upon temozolomide and quercetin treatment. Toxicol Appl Pharmacol (2013) 273(3):580–9. doi: 10.1016/j.taap.2013.10.003 24126416

[40] Shen G Liang S Xu Z Zhou L Xiao S Xia X. Downregulated expression of HSP27 in human low-grade glioma tissues discovered by a quantitative proteomic analysis. Proteome Sci (2010) 8 17:1–12. doi: 10.1186/1477-5956-8-17. 26. 20346134 PMC2858726

[41] Srinivasula SM Hegde R Saleh A Datta P Shiozaki E Chai J. A conserved XIAP interaction motif in caspase-9 and Smac/DIABLO regulates caspase activity and apoptosis. Nature (2001) 410:112–6. doi: 10.1038/35050012 11242052

[42] Kashkar H Haefs C Shin H Hamilton DSJ Salvesen GS Kronke M. XIAP-mediated caspase inhibition in hodgkin’s lymphoma-derived b cells. J Exp Med (2003) 198:341–7. doi: 10.1084/jem.20021279 PMC2194071 12874265

[43] Kashkar H Seeger JM Hombach A Deggerich A Yazdanpanah B Utermohlen O. XIAP targeting sensitizes Hodgkin lymphoma cells for cytolytic T-cell attack. Blood (2006) 108:3434–40. doi: 10.1182/blood-2006-05-021675 16868249

[44] Vladimirov YA Sarisozen C Vladimirov GK Filipczak N Polimova AM Torchilin VP. The cytotoxic action of cytochrome C/Cardiolipin nanocomplex (Cyt-CL) on cancer cells in culture. Pharm Res (2017) 34:1264–75. doi: 10.1007/s11095-017-2143-1 28321609

[45] Kao SJ Lee WJ Chang JH Chow JM Chung CL Hung WY. Suppression of reactive oxygen species-mediated ERK and JNK activation sensitizes dihydromyricetin-induced mitochondrial apoptosis in human non-small cell lung cancer. Environ Toxicol (2017) 32:1426–38. doi: 10.1002/tox.22336 27539140

[46] Irizarry Rovira AR Bennet BM Bolon B Braendli-Baiocco A Chandra S Fleurance R. Scientific and regulatory policy committee points to consider: Histopathologic evaluation in safety assessment studies for PEGylated pharmaceutical products. Toxicol Pathol (2018) 46:616–35. doi: 10.1177/0192623318791801 30092727

[47] Trotta AP Chipuk JE. Mitochondrial dynamics as regulators of cancer biology. Cell Mol Life Sci (2017) 74:1999–2017. doi: 10.1007/s00018-016-2451-3 28083595 PMC5419868

[48] Kadam CY Abhang SA. Serum levels of soluble fas ligand, granzyme b and cytochrome c during adjuvant chemotherapy of breast cancer. Clin Chim Acta (2015) 438:98–102. doi: 10.1016/j.cca.2014.08.012 25139496

[49] Liu Z Zhao X Zhang L Pei B. Cytochrome c inhibits tumor growth and predicts favorable prognosis in clear cell renal cell carcinoma. Oncol Lett (2019) 18:6026–6032. doi: 10.3892/ol.2019.10989 31788077 PMC6866253

[50] Al-Shakarchi W Alsuraifi A Abed M Abdullah M Richardson A Curtis A. Combined effect of anticancer agents and cytochrome c decorated hybrid nanoparticles for liver cancer therapy. Pharmaceutics (2018) 10(2):48. doi: 10.3390/pharmaceutics10020048 PMC6027273 29649145

