# Supplementary material for: Cytochrome C as a potential clinical marker for diagnosis and treatment of glioma
Source: Front Oncol. 2022 Sep 13;12:960787. doi: 10.3389/fonc.2022.960787 (PMC9513483; doi:10.3389/fonc.2022.960787)
Supplement: Supplementary file 1 [file Table_1.docx]

*Supplementary materials*

## Cytochrome C as a Potential Clinical Marker for Diagnosis and Treatment of Glioma

Rashmi Rana*^1^, Rohit Singh Huirem^1^, Ravi Kant^1^, Kirti Chauhan^1^, Swati Sharma^1^, M H Yashavarddhan^1^, Satnam Singh Chhabra^2^, Rajesh Acharya^2^, Samir Kumar Kalra^2^, Anshul Gupta^2^, Sunila Jain^3^, Nirmal Kumar Ganguly^1^

## *Author to whom correspondence should be addressed

Dr. Rashmi Rana

Email: [rashmi.rana@sgrh.com](mailto:rashmi.rana@sgrh.com) , [rana_aiims@yahoo.co.in](mailto:rana_aiims@yahoo.co.in)

**Supplementary tables**

**Supplementary Table-1: Details of patients involved in the studies.**

| **Grade I** | | | | |
| --- | --- | --- | --- | --- |
| **Sl. No.** | **Age** | **Gender** | **Sample** | **Microscopic findings** |
| 1 | 64 | F | Hemangioblastoma (Right cerebellum) | N.A. |
| 2 | 24 | F | Pilocytic astrocytoma | areas of cystic degeneration, interspersed vascular channels seen |
| 3 | 32 | F | Pilocytic astrocytoma | spongy stroma, microvascular proliferation, necrosis |
| 4 | 43 | M | pilocytic astrocytoma | Mild nuclear atypia, mitosis and oligodendroglial differentiation is seen, focal glomeroloid type of vascular proliferation seen. |
| 5 | 22 | F | pilocytic astrocytoma | Atypia is seen and no microvascular proliferation. |
| 6 | 49 | M | pilocytic astrocytoma | Necrosis seen and dystrophic calcification , reactive gliosis |
| **Grade II** | | | | |
| **Sl. No.** | **Age** | **Gender** | **Sample** | **Microscopic findings** |
| 1 | 35 | F | Tissue(astrocytoma) | Moderate nuclear pleomorphism |
| 2 | 19 | M | Tissue(Pilocytic astrocytoma) | Focal microvascular proliferation |
| 3 | 32 | M | Diffuse Astrocytoma (Right frontal region) | oligodendroglio differentiation seen, necrosis and increased mitosis seen. |
| 4 | 15 | M | OligoAstrocytoma (left frontal region) | Oligodendroglial pattern |
| 5 | 57 | F | right lateral ventricle central neurocytoma | few mitosis, necrosis |
| 6 | 50 | M | low grade with oligodendroglioma (1p19q codeletion suggested) | mild nuclear atypia, focal olgodendroglial pattern, necrosis |
| **Grade III** | | | | |
| **Sl. No.** | **Age** | **Gender** | **Sample** | **Microscopic findings** |
| 1 | 51 | M | High Grade Glioma (Anaplastic oligodendroglioma) | Anaplastic oligodendroglioma with focal astrocytic areas |
| 2 | 23 | M | Anaplastic Astrocytoma (left temporal region) | Large areas of necrosis |
| 3 | 45 | F | oligoastrocytoma (diffuse glioma) | 60% cells of total tumor is composed of monomorphic round oligodendroglial cells present in different sheets |
| 4 | 35 | M | diffuse glioma with anaplastic oligodendroglioma | mild atypia & few mitosis |
| 5 | 31 | F | Anaplastic astrocytoma | Moderate nuclear atypia and increased mitosis. |
| 6 | 46 | M | Anaplastic astrocytoma | Mild increase in mitosis, Necrosis seen. |
| **Grade IV** | | | | |
| **Sl. No.** | **Age** | **Gender** | **Sample** | **Microscopic findings** |
| 1 | 56 | M | Malignant astrocytoma | Vascular proliferation , large areas of necrosis, pseudopallisading and increased mitotic |
| 2 | 62 | M | Glioblastoma (IDH wild type) | High mitosis |
| 3 | 53 | F | Glioblastoma Multiforme | Astrocyte tumors with heterogenous morphology, large no. of gemistocytic cells |
| 4 | 54 | M | Astrocytoma (right frontal region) | focal areas of necrosis with pseudopallisading and microvascular proliferation |
| 5 | 58 | F | Glioblastoma Multiforme | high grade astrocytoma |
| 6 | 50 | M | Glioblastoma Multiforme (astrocytoma right parietal region) | Bizzare multinucleated giant cells with glial & spindle cells |
| **Control (Accidental cases)** | | | | |
| **Sl. No.** | **Age** | **Gender** | **Sample** | **Microscopic findings** |
| 1 | 62 | M | N.A. | N.A. |
| 2 | 32 | F | N.A. | N.A. |
| 3 | 58 | F | N.A. | N.A. |
| 4 | 65 | M | N.A. | N.A. |
| 5 | 57 | F | N.A. | N.A. |
| 6 | 19 | M | N.A. | N.A. |
